# Protocol of a Phase II Randomized, Multi-Center, Double-Blind, Placebo-Controlled Trial of S-Adenosyl Methionine in Participants with Mild Cognitive Impairment or Dementia Due to Alzheimer’s Disease

**DOI:** 10.14283/jpad.2023.55

**Published:** 2023-05-15

**Authors:** Sarah Holper, R. Watson, L. Churilov, P. Yates, Y. Y. Lim, K. J. Barnham, N. Yassi

**Affiliations:** 1 Department of Medicine - The Royal Melbourne Hospital, University of Melbourne, Parkville, VIC Australia; 2 Population Health and Immunity Division, The Walter and Eliza Hall Institute of Medical Research, 1G, Royal Parade, Parkville, VIC 3052 Australia; 3 Melbourne Medical School, University of Melbourne, Parkville, VIC Australia; 4 Department of Aged Care Services, Austin Health, Heidelberg, VIC Australia; 5 The Turner Institute for Brain and Mental Health, School of Psychological Sciences, Monash University, Monash, VIC Australia; 6 Florey Institute of Neuroscience and Mental Health, University of Melbourne, Parkville, VIC Australia; 7 Department of Neurology, Melbourne Brain Centre at The Royal Melbourne Hospital, University of Melbourne, Parkville, VIC Australia

**Keywords:** Alzheimer’s disease, S-adenosyl methionine, DNA methylation, tau hyperphosphorylation, dementia

## Abstract

**Background:**

S-adenosyl methionine (SAMe) is a pivotal metabolite in multiple pathways required for neuronal homeostasis, several of which are compromised in Alzheimer’s disease (AD). Correction of the SAMe deficiency that is characteristic of the AD brain may attenuate or prevent pathological processes driving AD-associated neurodegeneration including aberrant tau hyperphosphorylation and DNA hypomethylation.

**Objectives:**

The primary aim is to test the hypothesis that daily treatment with 400 mg oral SAMe for 180 days will lead to a greater reduction from baseline in plasma levels of p-tau181 compared to placebo in patients with mild cognitive impairment or dementia due to AD.

**Design, Setting, Participants:**

This is a phase II, randomized, multi-center, double-blind, placebo-controlled trial among 60 participants with mild cognitive impairment or dementia due to AD. Participants will be randomized in a 1:1 ratio to receive either SAMe or matching placebo, to be taken as an adjunct to their AD standard of care.

**Measurements and Results:**

The primary outcome is change in plasma p-tau181 concentration between baseline and following 180 days of treatment, which will be compared between the active and placebo group. Secondary outcomes are the safety of SAMe administration (incidence of serious adverse events), change from baseline in cognitive performance (as measured by the Repeatable Battery for the Assessment of Neuropsychological Status), and epigenetic changes in DNA methylation.

**Conclusion:**

Demonstration of effective and safe lowering of plasma p-tau181 with SAMe in this phase II trial would pave the way for an exciting field of translational research and a larger phase III trial.

**Electronic Supplementary Material:**

Supplementary material is available in the online version of this article at 10.14283/jpad.2023.55.

## Introduction

Plaques containing amyloid-β (Aβ), and neurofibrillary tangles consisting of hyperphosphorylated tau are the core pathological hallmarks of Alzheimer’s disease (AD). Overproduction and reduced clearance of Aβ are the key drivers of the disease, and Aβ toxicity and neurodegeneration are mediated through tau (1). Both Aβ and tau are also well-validated biomarkers of AD (2). As such, reduction of Aβ and/or tau levels is a prime target of therapeutic trials currently underway around the world. Aberrations in multiple molecular pathways contribute to the development and progression of AD pathology including tau hyperphosphorylation, DNA hypomethylation, and maladaptive neuroinflammation (3).

Despite advances in the understanding of the underlying mechanisms of AD, no effective disease modifying therapy exists. Results from clinical trials targeting single aspects of AD pathology, such anti-Aβ therapy, have been mixed. An attractive strategy would be to identify and target an ‘upstream’ aberration at the convergence of several pathological processes leading to the clinical manifestations of AD. This strategy is challenging given the diverse genetic, lifestyle, nutritional, and environmental risk factors that contribute to AD pathogenesis. None alone can account for the total prevalence of the disease; it is likely that disease manifestation occurs when multiple risk factors coalesce. For example, a genetic predisposition may become unmasked in the presence of age-related malnutrition, or changes in neuronal homeostasis.

S-adenosyl methionine (SAMe), the active form of the amino acid methionine, is the major metabolite in multiple metabolic pathways on which neuronal homeostasis depends. As a methyl donor, SAMe influences gene expression (via DNA methylation) and results in the synthesis of diverse products including neurotransmitters and phosphatases (4). SAMe is central to transsulfuration (which produces glutathione, the brain’s major antioxidant (5)), and the synthesis of neuroregulatory polyamines (which have anti-inflammatory properties (6) and are involved in controlling cell differentiation and proliferation (7)).

**Figure 1:**
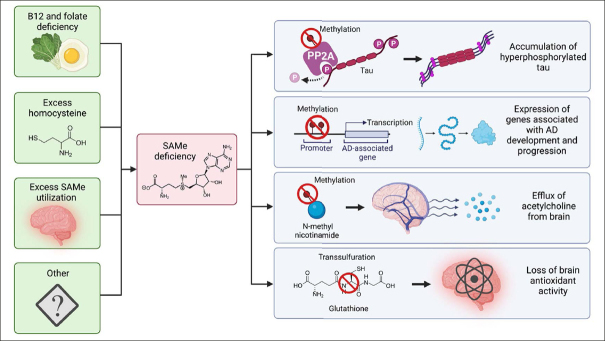
Causes and consequences of S-adenosyl methionine (SAMe) deficiency in AD

**Figure 2:**
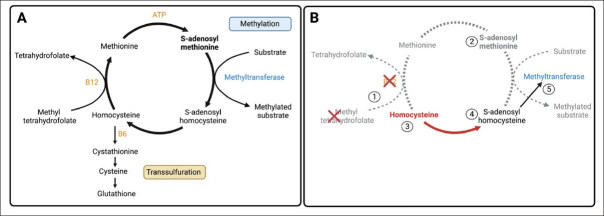
A) The methionine cycle. Methionine is activated by adenosine triphosphate (ATP) to form S-adenosyl methionine (SAMe). In the process of methylation, SAMe donates a methyl group to a substrate in a reaction catalyzed by a methyltransferase. The remaining product, S-adenosyl homocysteine (SAH) is converted to homocysteine. From here, homocysteine has two possible fates: transsulfuration to glutathione (a B6-dependent reaction) or remethylation to methionine (a vitamin B12-dependent reaction, using methyl tetrahydrofolate as a substrate). The latter produces much of the methionine required to generate new SAMe. B) Aberrations in the methionine cycle leading to quantitative and functional SAMe deficiency

Maintenance of these processes, and thus normal neuronal function, depends on an adequate source of SAMe. Significantly reduced SAMe concentrations have been identified in both the cerebrospinal fluid (8) and postmortem brain tissue (9) of patients with AD. The cause of SAMe depletion in the AD brain is likely multifactorial (see Fig. 1). Contributing factors may include B12 or folate deficiency (since methionine generation requires these nutrients), or excessive SAMe utilization in polyamine synthesis as part of a maladaptive chronic inflammatory response to tau pathology (10, 11). Elevated homocysteine, a sequela of B12 and folate deficiency, can also inhibit SAMe’s methylation function via negative feedback on methionine cycle enzymes (see Fig. 2 A and B) (12).

Several factors contribute to the SAMe deficiency characteristic of the AD brain including malnutrition, hyperhomocysteinaemia, excessive SAMe consumption in polyamine synthesis in the setting of chronic neuroinflammation, and other as-yet unknown mechanisms. SAMe deficiency accounts for multiple key pathological processes characteristic of AD. PP2A, the brain’s major phosphatase, is dysfunctional without methylation, allowing tau hyperphosphorylation to proceed unchecked. Loss of methylation of AD-associated genes (or their promotors) results in their expression. Brain acetylcholine levels fall owing to deficient SAMe-mediated methylation of N-methyl nicotinamide, which usually prevents the physiological net efflux of acetylcholine from the brain. Loss of SAMe-mediated transsulfuration depletes brain glutathione levels, impairing cerebral antioxidant activity and perpetuating neuroinflammation. AD: Alzheimer’s disease, P: phosphate, PP2A: protein phosphatase 2A

1. B12 or folate deficiency mean that homocysteine cannot be remethylated to methionine. These deficiencies may be quantitative (e.g. due to malnutrition) or functional (i.e. due to cerebral oxidative stress (25)). 2. SAMe levels fall due to lack of methionine (activation of dietary methionine is inadequate to meet the body’s SAMe needs; the body relies on de novo methionine synthesis generated via remethylation of homocysteine to sustain SAMe concentrations (24)). 3. Unable to be remethylated, homocysteine accumulates. 4. To achieve equilibrium, some excess homocysteine is converted back to SAH. 5. SAH is a potent inhibitor of methyltransferases with a much greater affinity for these enzymes than SAMe. As such, the low levels of SAMe remaining are unable to perform vital methylation reactions (i.e. functional SAMe deficiency).

Regardless of cause, SAMe deficiency exacerbates several key processes that underpin AD pathophysiology. SAMe deficiency increases the expression of genes involved in the onset and progression of AD, leading to increased Aβ production (13–16); compromises acetylcholine production and retention in the brain (17); impairs phosphatase function, resulting in accumulation of hyperphosphorylated tau (18); and hinders glutathione-driven quenching of reactive oxygen species (19) promoting a neuroinflammatory state.

There is preclinical evidence suggesting that SAMe supplementation improves cognition in multiple animal models of neurodegenerative conditions including the 3xTg mouse model of AD (17, 20, 21). In humans with AD, SAMe administration as a component of a nutraceutical supplement resulted in improvements in both cognitive function and dementia symptom severity, with excellent tolerability (22, 23).

Widespread predominantly non-prescription use of SAMe currently occurs mainly for depression, osteoarthritis and liver disease, although the quality of evidence supporting these uses is limited (24). Given SAMe’s critical role in multiple pathological processes central to AD development, a unique opportunity exists to examine the efficacy and safety of this cheap, readily available, oral medication in a clinical trial of patients with AD.

## Methods

**Figure 3:**
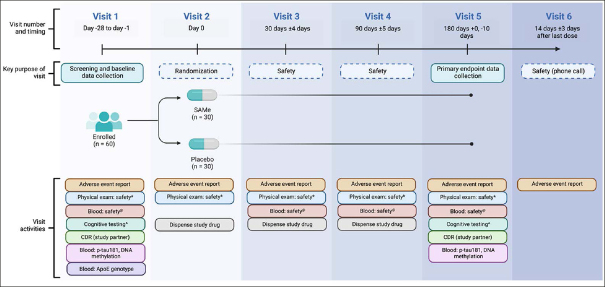
Summary of SAMe Study design. Participants will complete 6 study visits

This is a phase II, randomized, multi-center, double-blind, placebo-controlled trial to assess whether treatment with 400 mg of oral SAMe for 180 days will reduce plasma p-tau181 levels in patients with mild cognitive impairment or dementia due to AD. Key trial activities are summarized in Fig. 3. After baseline data collection, participants will be randomized in a 1:1 ratio to receive either 400 mg SAMe or matched oral placebo to be taken as an adjunct to their AD standard of care. Participants will be assessed for safety at 30, 90 and 180 days from treatment commencement, and at 14 days following treatment cessation. Efficacy will be determined by comparing data collected after 180 days of treatment with baseline assessments.

Each blue column summarizes the visit number, its timing and key purpose, and the study activities performed during the interaction. Visit 1 comprises obtaining informed consent, screening for eligibility, enrollment if suitable, and baseline data collection. Randomization and study drug dispensation occurs at visit 2, which constitutes ‘day 0’. Visits 3 and 4 involve safety assessments. Endpoint data collection occurs at visit 5. Visit 6 entails a follow-up telephone call to the participant for a final adverse event report. Visits will occur within an allowed range of days around the desired timepoint (indicated by ±). The endpoint data collection visit (visit 5) does not have an upper allowable bound: endpoint data is to be collected within the final 10 days of the participant’s 180 days taking the study drug. *:vital signs, body weight, physical examination; @: full blood examination, general biochemistry, liver function tests, coagulation studies, vitamin B12, folate, homocysteine; ^: Repeatable Battery for the Assessment of Neuropsychological Status, Standardized Mini-Mental State Examination, Cognitive Dementia Rating, California Verbal Learning Test 3rd Edition, Montreal Cognitive Assessment, Digit Symbol Substitution Test, Geriatric Depression Scale. ApoE: apolipoprotein E; CDR: Cognitive Dementia Rating; p-tau181: phosphorylated tau181; SAMe: S-adenosyl methionine.

The trial has been prospectively registered on australianclinicaltrials.gov.au (ACTRN12620000506998) and will be conducted within the estimand framework for the design and analysis of clinical trials. Briefly, the objective of the estimand framework is to align the clinical trial objectives with the study design, endpoints and analysis, in order to improve study planning and the interpretation of study results (26). The trial design and estimands include prespecified strategies for the conduct of the trial during the COVID-19 pandemic (27–29). The protocol is reported according to SPIRIT guidelines, modified through inclusion of estimand framework (30).

This study will be conducted in accordance with the Declaration of Helsinki and Council for International Organizations of Medical Sciences International Ethical Guidelines. The protocol has been approved by the local Human Research Ethics Committee.

### Aims and hypotheses

#### Primary aim

The primary aim is to test the hypothesis that treatment with 400 mg per day of oral SAMe for 180 days will lead to a greater reduction from baseline plasma levels of p-tau181 compared to placebo in patients with mild cognitive impairment or dementia due to AD.

#### Secondary aims

Secondary aims are to determine if, compared to placebo, treatment with 400 mg oral SAMe for 180 days will 1) maintain or improve performance as measured by the Repeatable Battery for the Assessment of Neuropsychological Status (RBANS), 2) result in epigenetic changes in DNA methylation, and 3) be safe.

### Exploratory aims

Exploratory aims are to evaluate the change from baseline that treatment with 400 mg oral SAMe for 180 days has, compared to placebo, on 1) blood total tau, amyloid-β, and neurofilament light levels, and 2) cognitive status as assessed by the Standardized MiniMental State Examination, Cognitive Dementia Rating, California Verbal Learning Test 3rd Edition, Montreal Cognitive Assessment, and Digit Symbol Substitution Test.

### Participants and setting

Community-dwelling adults aged 60 years and older with mild cognitive impairment or dementia due to AD who meet all other inclusion criteria will be eligible for recruitment, provided no exclusion criteria are met.

### Inclusion criteria

- Male and female individuals of at least 60 years of age at the time of the screening visit
- Diagnosis of mild cognitive impairment (31) or dementia (32) due to AD according to NIA-AA 2011 criteria
- Standardized mini-mental state examination score of at least 18 at the time of the screening visit
- Living in a community setting without continuous nursing care
- Can identify a reliable study partner with whom they have at least 3 weekly interactions to oversee study drug administration and participate in all clinic visits and some study procedures (including Cognitive Dementia Rating).
- If taking medications indicated for control of AD symptoms, on stable doses for at least 8 weeks prior to screening

### Exclusion criteria

- Any history of bipolar affective disorder
- A medical condition, other than AD, that may contribute to the individual’s cognitive impairment
- Stroke or transient ischemic attack in the past 12 months
- Clinically significant unstable psychiatric condition in the past 6 months
- Inability to swallow oral medications
- Other medical conditions which the investigator deems would limit the individual’s life expectancy to less than 6 months
- Concurrent use of anti-depressant medication
- Participation in another interventional clinical trial, or intake of another investigations drug within 4 weeks or 5 half-lives (whichever is longer)

### Study Estimands

#### Primary estimand

1. Population: all included participants.
2. Individual level outcome measure: percentage change in plasma p-tau181 concentration (comparing baseline to 180 days). P-tau181 levels will be measured using the single-molecule array (Simoa) technique according to the manufacturer’s instructions (Quanterix Corp., US).
3. Population level outcome measure: difference in means between intervention and control groups adjusted for baseline p-tau181 level.

##### Intercurrent event strategy

Intercurrent events describe circumstances occurring after randomisation that preclude outcome observation or affect its interpretation. We have pre-specified possible intercurrent events and devised strategies for their handling:

1. Inadequate study drug adherence: Given that establishing biological efficacy is the primary objective of the trial, all participants who achieve at least 80% study medication compliance and provide a 180-day blood sample will be included in the primary efficacy analysis (principal stratum strategy). As a supplementary estimand, we will also perform a dose-response analysis in all participants who provide a blood sample within the required time period (regardless of medication compliance)
2. COVID-19 infection: The main analysis of the primary estimand will include participants with COVID-19 infection. As a first sensitivity analysis for the primary estimand, we will perform the primary analysis excluding participants with confirmed COVID-19 infection before or during the study.
3. Inability to collect sample for primary outcome: As a supplementary analysis for the primary estimand, the missingness at random assumption will be evaluated for any patient or system related intercurrent events preventing the collection of the primary outcome. A sensitivity analysis using a pattern mixture model will be undertaken.

#### Secondary estimand — cognitive improvement

1. Population: all included participants
2. Individual level outcome measure: total scaled RBANS change, comparing treatment with 180 days of 400 mg oral SAMe to placebo
3. Population level outcome measure: difference in means between intervention and control groups. Difference between the groups will be analyzed adjusted for baseline RBANS.

The RBANS is a multidimensional screening battery of cognitive tests useful in a variety of populations including AD, mild cognitive impairment, and other cognitive disorders (33). It has two alternate forms, and yields five index scores; Immediate Memory, Visuospatial/Constructional, Language, Attention, and Delayed Memory.

##### Intercurrent event strategy

1. Intercurrent illnesses or medication changes (including addition of sedating medications or unavailability of regular symptomatic AD medications): This estimand will be analysed in participants who undergo follow-up RBANS assessment at 180 days (+0, −10 days) and who have taken at least 80% of the study medication, and where there have been no intercurrent events blindly adjudicated as affecting cognition (principal stratum strategy)

#### Secondary estimand — epigenetic change

1. Population: all included participants
2. Individual level outcome measure: DNA methylation changes
3. Population level outcome measure: will be described using bioinfomatic techniques

##### Intercurrent event strategy

1. This estimand will be analysed in participants who undergo follow-up RBANS assessment at 180 days (+0, −10 days) and who have taken at least 80% of the study medication (principal stratum strategy)

#### Secondary estimand – safety

1. Population: all included participants
2. Individual level outcome measure: presence or absence of serious adverse events (SAEs)
3. Population level outcome measure: Difference in proportion of participants with SAEs present between the intervention and control groups

##### Intercurrent event strategy

1. Inadequate study drug adherence: This estimand will be analysed in participants who have taken at least 1 dose of study medication (principal stratum strategy).
2. COVID-19 infection: COVID-19 related SAEs will be included in the analysis for this estimand, as for SAEs related to any other intercurrent events. Given the unique risk of SAEs due to the potential for increased incidence of COVID-19 over the study period, we will also perform a sensitivity analysis where COVID-19 related SAEs will be excluded from the analysis.

### Sample size

Allowing for potential participant attrition, the total sample size for the study is 60 (30 per arm). Sample size calculations assumed a two-tailed alpha of 0.05 and a similar baseline standard deviation between the two arms. A total sample size of 52 participants (26 per arm) would yield 80% power to observe a large treatment effect (Cohen’s d 0.8). This sample size estimation is conservative in the sense that including the baseline p-tau181 concentration in the final ANCOVA model would increase the power.

### Study procedure

#### Screening and enrollment

Informed consent will be obtained from both participants and their study partner. In cases where the participant lacks capacity to provide informed consent, their medical treatment decision maker will perform this role. Eligibility assessment will then occur. Evidence for criteria fulfilment will be based on patient and caregiver report, and documentation from medical specialists obtained prior to the visit. A Standardized Mini-Mental State Examination will be performed during the visit, prior to enrolment, to confirm a score of at least 18. Following enrolment, participants will undergo an initial safety assessment comprising both a physical examination and blood sampling. Baseline data collection will then occur.

#### Randomization

Participants will be randomized within 28 days of enrollment. The results of safety blood tests will be reviewed for clinically significant abnormalities prior to randomization. A central randomization schedule has been generated by an independent statistician who is a part of the data center at the Melbourne Brain Centre at Royal Melbourne Hospital, University of Melbourne. The randomization schedule has been programmed into an electronic case report form (eCRF) system, housed on a RedCAP platform (34, 35). Participants will be randomized in a 1:1 ratio to receive either 400 mg SAMe or matched placebo orally once per day for 180 days. Randomization will be stratified based on age at enrolment (<70 years vs ≥70 years) to minimize baseline imbalance between the treatment groups.

#### Blinding

This is a double-blind study. Treatment allocation will be coded to prevent unblinding. The unblinded randomization schedule will be kept as a secure spreadsheet accessible only to the study manager (employed by the sponsor) and by the data center coordinating the eCRF. If a participant’s intervention assignment is unblinded, the investigator will notify the sponsor within 24 hours.

### Intervention

Participants will be instructed to take the assigned treatment once a day for a period of 180 days as an adjunct to their Alzheimer’s disease standard of care. No dose reductions will be considered.

#### Dose justification

SAMe is widely available over the counter as a commercial product in tablet or capsule form. Most formulations contain 200 or 400 mg of SAMe per pill. Pure SAMe pills are distributed by over a dozen international manufacturers (Supplementary Table 1). Conversely, Australian consumers only have access to SAMe in combination with other active ingredients. The Australian Register of Therapeutic Goods lists 4 SAMe-containing supplements: 3 of these combine SAMe with B-vitamins, another contains 8 additional herbs and nutraceuticals (Table 1). The per-pill SAMe content ranges from 26.3 mg to 400 mg. All products are recommended to be dosed once daily with total daily doses between 157.8 mg and 1200 mg, with a median and mode of 400 mg (mean 472.2 mg). A review of pure SAMe products available internationally also identified 400 mg to be the most frequent recommended total daily dose (Supplementary Table 1). As such, we have selected 400 mg orally (pure form) once daily as the dose and route for this phase II trial.

**Table 1:** SAMe-containing products listed by the Australian Register of Therapeutic Goods

**Brand name**	**Manufacturer**	**SAMe content per pill (mg)**	**Other active ingredients**	**Administration**	**Recommended total daily dosage in mg (number of pills)**
Orthoplex SAMe	Bio Concepts Pty Ltd	200	Pyridoxine, riboflavin, zinc	Oral, once daily	200–600 (1–3)
Orthoplex SAMe	Bio Concepts Pty Ltd	400	Pyridoxine, riboflavin, zinc	Oral, once daily	400–1200 (1–3)
ArthClear	BlueSkyGreenEarth Herbs Pty Ltd	26.3	Colecalciferol, curcumin, cysteine, glucosamine, manganese, quercetin, selenium, Boswellia serrata	Oral, once daily	105.2–157.8 (4–6)
SAMe 400 Complex	Nutrition Care Pharmaceuticals Pty Ltd	400	Cyanocobalamin, folic acid, pyridoxine, riboflavin, zinc	Oral, once daily	400–800 (1–2)

### Cognitive and psychological testing

Cognition will be evaluated using 7 instruments: the Standardized Mini-Mental State Examination (36, 37), Cognitive Dementia Rating (38), California Verbal Learning Test 3rd Edition (39), Montreal Cognitive Assessment (40), Digit Symbol Substitution Test (41), and the Geriatric Depression Scale (42).

### Independent Data Safety Monitoring Board (DSMB)

To compare the safety of SAMe versus placebo, interim safety analyses will be performed following the collection of the final visit data in the first 20 participants, and after 40 participants. The endpoint for this analysis will be the proportion of participants with SAEs in each arm of the trial. If there are concerns about the safety of participants, the independent DSMB will make a recommendation to the trial executive committee regarding continuing, stopping or modifying the trial. The Haybittle-Peto procedure for generating early stopping boundaries will be used (43). A recommendation for early termination due to safety reasons will be considered by the independent DSMB if the Haybittle-Peto boundary (p=0.001, Z=3) at a given interim analysis is crossed.

No interim analysis for efficacy or futility is planned.

### Statistical analysis

All outcomes and analyses are prospectively categorized as primary, secondary or exploratory. Differences in all endpoints between the 2 arms of the study will be tested independently at the two-tailed 0.05 level of significance. All estimates of treatment effects will be presented with 95% confidence intervals. The primary endpoint will be tested using an ANCOVA model with treatment arm as an independent variable and baseline blood p-tau181 concentration as a covariate. Between group differences in secondary and exploratory outcomes will be assessed using regression models as appropriate to the response outcome distribution using baseline values of the respective outcomes as adjustment covariates. The detailed analysis will be prespecified in a formal statistical analysis plan which will be finalised prior to the study database lock.

No formal adjustments will be undertaken to constrain the overall type I error associated with the secondary and exploratory analyses. Their purpose is to supplement evidence from the primary analysis to characterize the treatment effect more fully. Results from the secondary analyses will be interpreted in this context.

Given that establishing biological efficacy is the primary objective of the trial, according to the pre-specified estimand, all participants who achieve at least 80% study medication compliance and provide a 180-day blood sample will be included in the primary efficacy analysis. All participants who provide both baseline and end of treatment blood samples will be included in a secondary dose-response analysis to account for potential early study withdrawals or poor compliance.

## Discussion

Present in all living cells, SAMe is the active form of the amino acid methionine. Since its structural elucidation in 1952 (44), SAMe has become recognized as the major metabolite in multiple metabolic pathways on which neuronal homeostasis depends. In the brain, SAMe is the only methyl donor for numerous reactions catalyzed by methyltransferases with substrates including DNA, neurotransmitters, signal transduction systems, and membrane phospholipids (4). SAMe’s role in transsulfuration produces glutathione, the brain’s major antioxidant (5). SAMe is required for the synthesis of the neuroregulatory polyamines spermidine and spermine, which have anti-inflammatory properties (6) and are involved in controlling cell differentiation and proliferation (7).

Clearly, SAMe’s cellular functions are of direct relevance to the prevention of neurodegeneration and its depletion, as seen in the AD central nervous system, would be expected to carry widespread deleterious sequelae. In this trial, we will explore the effect of SAMe supplementation on tau phosphorylation and epigenetics, via measurement of plasma p-tau181 and DNA methylation, respectively, in patients with AD.

Tau hyperphosphorylation: Neurofibrillary tangles comprising hyperphosphorylated tau are a hallmark of AD pathology. Tau’s degree of phosphorylation is a result of the balance between kinase and phosphatase activity. Protein phosphatase 2A (PP2A) accounts for over 70% of the brain’s phosphatase activity (45). PP2A’s stability and enzymatic activity rely on SAMe-mediated methylation of its catalytic subunit. Both the concentrations and activity of PP2A are significantly reduced in the AD brain (46, 47). Owing to diminished PP2A activity, SAMe depletion would be expected to favor the accumulation of phosphorylated tau (48).

Pathological DNA hypomethylation: Gradual, global DNA hypomethylation is a feature of normal aging, though is accelerated in the AD brain (49) and can lead to overexpression of genes involved in the onset and progression of AD. SAMe depletion in AD animal models and in vitro has been shown to perturb DNA methylation with resultant increases in the expression of Amyloid Beta Precursor Protein (APP), Presenilin-1 (PS1) and Beta-Secretase 1 (BACE1) genes, stimulating Aβ production and neurodegeneration (13–16). A genome-wide analysis of DNA enhancer methylation in neurons from AD brains identified a large cluster of significantly hypomethylated enhancers in the DSCAML1 gene that targets BACE1 (50). Hypomethylation at this site was associated with an upregulation in BACE1 gene products and corresponded to an increase in amyloid plaque load, neurofibrillary tangle density, and a higher severity of cognitive symptoms. In a transgenic model of AD, SAMe administration reversed DNA hypomethylation, including at the BACE1 promoter (51), leading to decreased Aβ accumulation and cognitive improvements.

There is preclinical evidence suggesting that SAMe supplementation improves cognition in multiple animal models of neurodegenerative conditions including the 3xTg mouse model of AD (17, 20, 21). In a model of SAMe depletion achieved via folate deprivation, subsequent SAMe supplementation restored acetylcholine concentrations and cognitive performance to levels seen in the presence of folate, and reduced aggressive behavior (17). SAMe also reversed PS1 over-expression, leading to decreased gamma-secretase activity, and Aβ levels (20). In 3xTg-AD mice, dietary SAMe supplementation reduced hippocampal Aβ and phosphorylated tau, and delayed the accumulation of extracellular Aβ, demonstrating SAMe’s ability to modulate the time course of AD neuropathology (21, 23).

In humans with AD, SAMe has only been tested as part of a combined nutraceutical supplementation, rather than in its pure form. In a phase I trial of community-dwelling adults with mild to moderate AD, daily supplementation with a 6-ingredient nutraceutical formulation (NF) including 800 mg of SAMe resulted in statistically significant improvements in both cognitive function and severity of behavioural and psychiatric symptoms of dementia (BPSD) within 6 months, with improvement maintained for 28 months (52). The improvements in BPSD after 3 and 6 months were equivalent to published efficacies for donepezil and in excess of those for galantamine. In another phase I trial among adults with moderate to severe AD, the same formulation significantly delayed cognitive decline compared to placebo (53). Unlike the placebo group, those receiving the NF maintained baseline cognitive performance on the Dementia Rating Scale (DRS) for 6 months and displayed only a 20% decline by 3 months in the CLOX1 task (a measure of executive function), compared to the 72% decline seen in the placebo group. When extended to a phase II trial including over 100 patients with AD, those taking the NF not only maintained their cognitive performance (as measured by DRS and CLOX1) after 3 months, but in fact achieved improved scores, with excellent tolerability (22).

All considered, these data strongly suggest that SAMe depletion contributes to multiple pathophysiological processes observed in the AD brain, including the unsilencing of genes involved in AD development and progression, the depletion of acetylcholine, and the inability to appropriately dephosphorylate tau. The reversibility of these deleterious sequelae by SAMe supplementation in animal models of AD warrants further exploration in humans, where similar results could revolutionize the treatment of AD.

## Electronic Supplementary Material

Supplementary material, approximately 23.5 KB.
