# Supplementary material for: Protocol of a Phase II Randomized, Multi-Center, Double-Blind, Placebo-Controlled Trial of S-Adenosyl Methionine in Participants with Mild Cognitive Impairment or Dementia Due to Alzheimer’s Disease
Source: J Prev Alzheimers Dis. 2023 May 15:1–10. Online ahead of print. doi: 10.14283/jpad.2023.55 (PMC10186290; doi:10.14283/jpad.2023.55)
Supplement: Supplementary file 1 — Supplementary material, approximately 23.5 KB. [file 42414_2023_55_MOESM1_ESM.docx]

**SUPPLEMENTARY MATERIAL**

Supplementary Table 1: Pure SAMe products available internationally*
All products contained either 200 mg or 400 mg of SAMe. Recommended administration frequency ranged from once to 6 times daily. For any single administration, the dose ranged from 200 mg to 800 mg. Recommended total daily dose ranged from 200 mg to 1600 mg. The most frequent recommended total daily dose (mode) was 400 mg, mean 779.7 mg, median 800 mg.

| Manufacturer | SAMe content per pill (mg) | Administration (one pill unless otherwise stated) | Recommended total daily dose in mg (number of pills) |
| --- | --- | --- | --- |
| Doctor’s Best | 200 | Oral, twice daily | 400 (2) |
| Doctor’s Best | 400 | Oral, once daily | 400 (1) |
| Life Extension | 200 | Oral, 2-6 times daily | 400-1200 (2-6) |
| Life Extension | 400 | Oral, 1-3 times daily | 400-1200 (1-3) |
| NutraLife | 200 | Oral, once daily | 400 (2) |
| Source Naturals | 400 | Oral, 1-3 times daily | 400-1200 (1-3) |
| California Gold Nutrition | 400 | Oral, once daily | 400 (1) |
| Natural Factors | 200 | Oral, twice daily | 200-1600 (1-8) |
| Jarrow Formulas | 200 | Oral, 1-2 times daily | 200-400 (1-2) |
| Jarrow Formulas | 400 | Oral, once daily | 400 (1) |
| NOW Foods | 200 | Oral, 2-4 times daily | 400-800 (2-4) |
| NOW Foods | 400 | Oral, 2-4 times daily | 800-1600 (2-4) |
| Natrol | 400 | Oral, once daily | 800 (2) |
| Piping Rock | 400 | Oral, 1-3 times daily | 400-1200 (1-3) |
| Pure Encapsulation | 200 | Oral, 1-2 times daily | 200-400 (1-2) |
| Swanson ULTRA | 400 | Oral, 1-4 times daily | 400-1600 (1-4) |
| Douglas Laboratories | 200 | Oral, 1-2 times daily | 200-400 (1-2) |
| Bioclinic Naturals | 200 | Oral, 1-4 pills twice daily | 200-1600 (2-8) |
| Progressive Laboratories | 400 | Oral, once daily | 400 (1) |
| Protocol for Life Balance | 200 | Oral, 2-4 times daily | 400-1600 (2-4) |
| Protocol for Life Balance | 400 | Oral, 2-3 times daily | 800-1200 (2-3) |

*This data set was generated by including all pure SAMe products available for sale on 3 large international supplement distributor websites: megavitamins.com, iHerb.com, supplementfirst.com.

Supplementary Table 2

COVID-19 impact. In response to the COVID-19 pandemic, contingency plans were developed to mitigate the potential impact of COVID-19 on the safety of participants and investigators, and on trial outcomes. Supplementary Table 2 details these considerations.

| COVID-19-related factor | SAMe Study impact and contingency plan |
| --- | --- |
| COVID-19 testing | In line with government guidelines, participants or potential participants who undergo COVID-19 testing are required to isolate while awaiting a result. Baseline visits will be postponed in these participants until a negative test can be confirmed. Study activities have been revised to ensure most of the study can be conducted remotely via telehealth. Study activities which require face-to-face assessment (either on-site or in the participant's home) will be performed within the relevant allowable period (which is generally longer than testing turnaround times). |
| COVID-19 infection | There has been some early evidence that COVID-19 infection has some effects on the central nervous system, with some studies suggesting a specific effect on tau homeostasis, and particularly an increase in tau phosphorylation [1]. However, there is yet to be a demonstration that COVID-19 infection increases levels of circulating pTau181, and based on this, participants with confirmed COVID-19 will be eligible for recruitment and continuation in the study (once they have recovered). Participants who miss the primary estimand time window due to COVID-19 infection (or other intercurrent events) will be excluded from the primary analysis using a principal stratum approach. In addition, a sensitivity analysis for the primary estimand will be performed excluding participants with confirmed COVID-19 at any point before or during the study. |
| Quarantine and travel limitations | The study has been designed to allow participation in all study assessments from the participants' home. This will involve either telehealth consultations and/or home visits by qualified study personnel who will comply with all current PPE requirements. This will minimize the potential of quarantine and travel limitations on study assessments. |
| Site closures | Loss to follow-up due to site closure is expected to be minimal given that site selection and initiation occurred once the pandemic was already underway, and the COVID-19 contingency plan was discussed with all potential sites. In the unlikely event of site closure, the primary site (WEHI) will be able to collect the primary endpoint data to inform the primary estimand. |
| Interruption to Supply Chain of Investigational Product or Participant’s Other Medications | There may be some delays in IP kit production and transport to sites because of COVID-19 restrictions. In the event of delays or interruptions to the IP supply chain, sites will be instructed to randomize participants only if there is sufficient IP available at the given site. Recruitment will be suspended if IP supplies are exhausted at a site until IP has been resupplied. Regarding concurrent medications, participants are required to be on a stable dose of symptomatic AD medications (e.g. donepezil) for 8 weeks prior to randomization, and for the duration of the study. This requirement will be maintained. The secondary (cognitive endpoint) will be analyzed only within the subgroup of participants where there has been no change in symptomatic medication (due to availability or intercurrent events) throughout the study period, or where no other intercurrent event adjudicated as potentially affecting cognition has occurred |
| Stopped Enrolment | COVID-19 infection surges and restrictions may lead to temporary site-specific recruitment reductions, delays, suspension, and potentially termination of recruitment at specific sites. The study is being conducted at several sites, with the main site being a research institute (not a health care facility). The risk of termination of recruitment across the entire trial due to COVID-19 is low. The need for unplanned interim analysis for futility due to COVID-19 is expected to be unlikely. |
| Delayed Assessments | There is a risk of inability to conduct some study assessments during the allowable study visit time frame due to COVID-19 testing or confirmed infection as well as due to other COVID-19 related intercurrent events. For the primary estimand, the analysis will be conducted only in participants in whom the primary endpoint is collected within the allowable range of days and who have taken ≥80% of the study medication. A similar approach will be used for the epigenetic change secondary estimand. For the cognitive secondary estimand, missing data due to COVID-19 related intercurrent events will be treated as missing at random subject to the satisfiability of the relevant assumption. Auxiliary variables that may potentially contribute to verifying this assumption will be collected, particularly, age. This is expected to be less likely given that the trial has been designed to allow telemedicine visits for the cognitive outcomes. |
| Missed Visit Assessments | As above (see ‘delayed assessment’). |
| Stopped Drug due to Safety Concerns | There are no known adverse effects of SAMe in the setting of COVID-19 infection. Therefore, we do not anticipate that study drug cessation due to COVID-19 infection will be common. |
| Discontinuing Participants due to Infection | While COVID-19 presents a unique risk of AEs / SAEs due to the potential for increased incidence over the study period, we will include COVID-19 related deaths and SAEs in the safety estimands, as for any other intercurrent causes of SAEs/AEs. We will undertake sensitivity analysis where SAEs due to COVID-19 will be excluded from the analysis |
| Alternative Collection of Specimens | Telehealth and home-visit assessments will allow ongoing collection of data, both biological specimens and cognitive assessments, during the COVID-19 pandemic. |
| Alternative Data Collection | As above (see ‘Alternative Collection of Specimens’). |

**REFERENCES**

1. Gopalakrishnan, J.; Ramani, A.; Mueller, L.; Ostermann, P. N.; Gabriel, E.; Pranty, A. I.; Mueller-Shiffmann, A.; Mariappan, A.; Goureau, O.; Gruell, H., SARS-CoV-2 targets cortical neurons of 3D human brain organoids and shows neurodegeneration-like effects. *bioRxiv* **2020**, 0.15252/embj.2020106230.
